# Towards a Chemiresistive Sensor-Integrated Electronic Nose: A Review

**DOI:** 10.3390/s131014214

**Published:** 2013-10-22

**Authors:** Shih-Wen Chiu, Kea-Tiong Tang

**Affiliations:** Department of Electrical Engineering, National Tsing Hua University/No. 101, Sec. 2, Kuang-Fu Road, Hsinchu 30013, Taiwan; E-Mail: swchiu.nthu@gmail.com

**Keywords:** metal-oxide semiconductor, conductive-polymer, sensing front-end, electronic nose signal processing chip, electronic nose system-on-chip (SoC)

## Abstract

Electronic noses have potential applications in daily life, but are restricted by their bulky size and high price. This review focuses on the use of chemiresistive gas sensors, metal-oxide semiconductor gas sensors and conductive polymer gas sensors in an electronic nose for system integration to reduce size and cost. The review covers the system design considerations and the complementary metal-oxide-semiconductor integrated technology for a chemiresistive gas sensor electronic nose, including the integrated sensor array, its readout interface, and pattern recognition hardware. In addition, the state-of-the-art technology integrated in the electronic nose is also presented, such as the sensing front-end chip, electronic nose signal processing chip, and the electronic nose system-on-chip.

## 1. Introduction

Olfaction is one of the five major human senses (vision, hearing, olfaction, taste, and touch). The sense of smell is the most mysterious and complex sense; a particular smell can trigger a series of memories in people. In 2004, Axel and Buck won the Nobel Prize in Physiology or Medicine for their research on “odorant receptors and the organization of the olfactory system” [1], which shows the interest and value of the research on olfaction. Artificial olfaction (also called an electronic nose or e-nose) is a biomimetic olfactory system [2] that can replace well-trained experts in dangerous work, or surpass the limits of their abilities. Recently, artificial olfaction has been developed for numerous industry applications [3], such as indoor air-quality monitoring [4], medical care [5–7], customs security [8], food quality control [9–15], environmental quality monitoring [16–18], military applications [19], and hazardous gas detection [20,21]. The earliest artificial olfaction device can be traced back to 1961, when Moncrieff proposed a mechanical artificial nose [22]. The first electronic nose was developed by Wilkens, Hatman [23], and Buck [24] in 1964. The concept of an electronic nose using a chemical sensor array system for odor classification was proposed by Persaud [25] in 1982. The term “electronic nose” was coined at a meeting in 1988 [26]; the first meeting focusing specifically on the electronic nose was held in 1991 [27]. Thus the electronic nose has now been in development for over 50 years.

Compared to traditional gas analysis methods, such as gas chromatography mass spectrometry (GC-MS) and Fourier transform infrared (FT-IR) spectrometry, the electronic nose has the potential to be small, fast, and inexpensive [28,29], which are great benefits for a gas identification mechanism. Furthermore, the electronic nose is suitable for non-expert users and easily applicable to daily life [30]. Like the mammalian olfactory system, non-selective sensors are used as mammalian receptors to generate a specific pattern, and then the pattern for each odor is identified. The method of odor identification is not designed for identifying the individual chemicals responsible for odors. Conventional odor analysis methods often become more incontrollable and less reliable as the complexity of constituent chemicals increases. Compared to conventional odor analysis methods, the electronic nose has a simple structure to benefit from the reduced size and cost. However, commercial electronic noses are large, non-portable, lab-type instruments. Commercial electronic noses can be divided into various sensor types [31], including conductive sensors (metal-oxide semiconductor, MOX; conductive polymer, CP) [32–39], piezoelectric sensors (quartz crystal microbalance, QCM; surface acoustic wave, SAW) [40–43], MOS field-effect transistor (MOSFET) sensors [44,45], optical sensors [46,47], and spectroscopy-based sensors (mass spectrum, MS; ion mobility spectroscopy, IMS) [48–50]. Table 1 lists commercially available electronic nose instruments; most are priced from US$20,000 to $100,000. The electronic noses of most manufacturers are realized by fixing gas collectors and detecting devices to personal or notebook computers, and weigh between 15 and 75 kg. Therefore, the high price and non-portability mean that electronic noses are only affordable by companies, organizations, and research institutions. With the rise of intelligent electronic products, cell phones have developed increasingly powerful functions; therefore, a method for using smart phones to develop an electronic nose system has attracted a considerable amount of interest. The design of electronic noses and general electronic products is closely related to the pursuit of inexpensive micro-scale devices, high compatibility with other consumer electronics and low power consumption. In [51], the authors predicted that the price of an electronic nose will be $1 by 2020. In summary, electronic nose products are available on the market, and currently provide solutions to a wide range of tasks in various areas. However, the electronic nose has not achieved its full potential as a commercial device; the bulky size and high price restrict its applications in daily life. Fortunately, the appearance of new sensing materials, development of fabrication technologies, and evolution of data processing methods offer the possibility of creating the next generation of electronic noses.

Research is being conducted on a portable electronic nose [6,8,55–61]; the development of a small and inexpensive portable electronic nose remains in the laboratory stage. In 1994, Hatfield proposed fabricating components of an electronic nose by using advanced integrated circuit (IC) technology to reduce the size and power consumption [62]. An IC is a set of electronic circuits on a small chip of semiconductor material. ICs can be made far more compact than independent components can be. The price of IC can be relatively very low with mass production. Currently, IC technology is an inextricable component of modern life that has made economical computers, mobile phones, and other digital appliances possible. A benefit of IC technology advancements is the continued discussion and emphasis on the fabrication of electronic noses using ICs. For example, the application-specific IC (ASIC) is specifically designed for odor classification based on electronic nose data [63]. Micro Electro Mechanical Systems (MEMS)-level metal-oxide semiconductor (MOX) sensor arrays integrate with the sensory interface circuit in a single chip [64]. The electronic nose chip acquires the sensor data and processes it directly, connecting with the off-chip sensor array [65]. These studies indicate the potential and benefits of implementing the electronic nose through advanced IC technology. For a highly integrated electronic nose, a conductive sensor is highly applicable to the integration of IC because of its simple electrical properties and interface circuit. It can be divided into two types, MOX [64] and conductive polymer (CP) [65], which display various resistance values when exposed to odors.

This study focuses on state-of-the-art technology to implement an inexpensive, miniature chemiresistive sensor-integrated electronic nose. Sections 1 and 2 present the introduction and an overview of portable electronic nose systems based on chemiresistive sensors, respectively. Section 3 presents the integrated chemiresistive sensor technology. Section 4 presents the chemiresistive sensor interface. Section 5 presents the highly integrated electronic nose system and specific electronic nose ICs. Finally, Section 6 presents a conclusion and discussion.

## 2. Portable Chemiresistive Sensors Based Electronic Nose System

**Figure 1.**
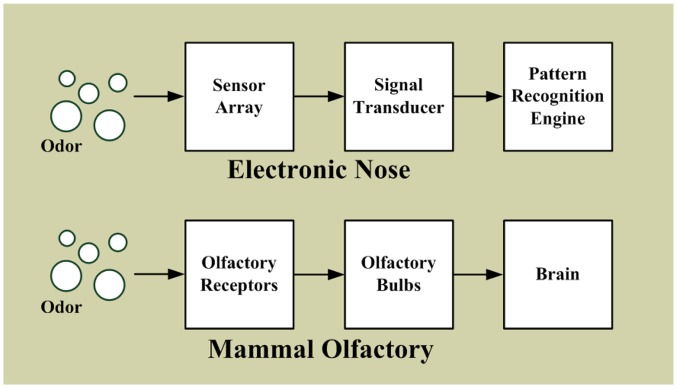
The basic gas identification system blocks: an electronic nose and a mammal olfactory.

Inspired by the structure of mammalian olfaction [2,29], electronic nose systems are primarily composed of a sensor array, a signal transducer, and a pattern recognition engine, as shown in Figure 1 [66–69]. In mammalian olfactory systems, olfactory receptor cells are vital sensory cells for sensing odors. In the nasal cavity, there are 6-10 million olfactory receptor cells [68,70]; the human genome contains approximately 900 different olfactory receptor genes, and the mouse genome contains approximately 1,300 [71]. When an odor enters the cavity, the olfactory signals activate in the olfactory receptor cells. The olfactory bulbs collect and convert the olfactory signals into neurological signals, and subsequently send these signals to the brain for odor identification. Although numerous types of olfactory receptor cells exist, the odor identification system of mammals is not based on one type of receptor cell for one specific odor. The olfactory system of mammals does not detect an odor by using just one sensor, but scents are sensed and recognized according to an array of multiple receptor cells, and each combination represents sensing a different odor that represents an odor “fingerprint”. Numerous permutations and combinations exist, enabling mammals to distinguish many different odors. A similar system is adopted for the electronic nose. The non-selected sensors forming the sensor array are used to detect odor, generating and identifying the odor “fingerprint”. However, the number of sensors for most electronic systems is limited; sensors typically numbering between several and several tens are often chosen depending on the application [52].

**Figure 2.**
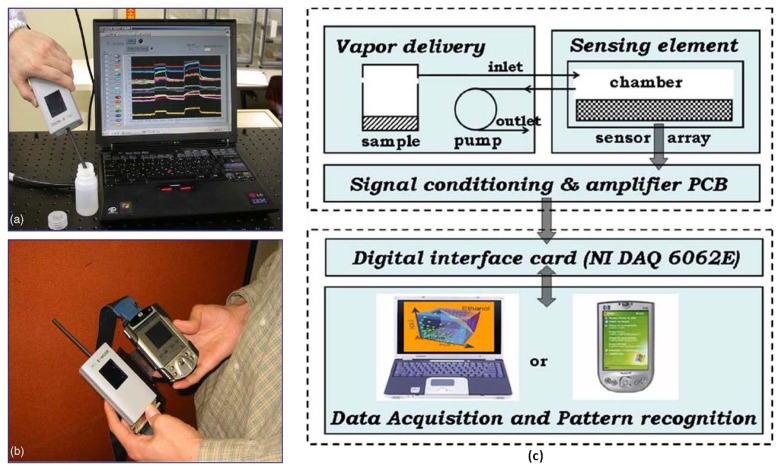
The portable electronic nose system consists of the hand-held sensing module and the personal digital apparatus: (**a**) laptop computer, and (**b**) PDA; (**c**) the block diagram of the system. Reprinted with permission from [81].

Low-cost electronic systems have been fabricated and applied in numerous ways based on the artificial olfaction structure and chemiresistive sensors. For example, the methodology of signal processing from the outputs of commercial FIGARO^®^ sensors has been proposed for food quality testing [72,73]. Using chemiresistive sensors, a platform was built for odor measurement, evaluated based on data and feature analysis, and applied in the development of an odor classification system [74–78]. Certain low-cost electronic nose systems are designed for indoor air-quality monitoring [79]; furthermore, combining them with a wireless module enables real-time online odor detection [80]. For individuals, portable electronic nose systems comprise the sensing module, the data acquisition board, and a personal computer (PC) for data analysis [6,8,57–58]. However, the PC could be replaced with a personal handheld electronic device, such as a personal digital assistant (PDA) [81], as shown in Figure 2. In this study, the portable electronic nose system consisted of a hand-held sensing module and a PDA embedded with a support vector machine algorithm in MatLab. The handheld sensing module comprised three major parts: gas delivery components, a sensor array chip, and signal conditioning circuitry. Because the 16 sensors are integrated on a single Si substrate, the sensor array is miniaturized to a size of 14 × 34 mm^2^; this is the primary reason that the sensing module can be reduced to a handheld size. Finally, the capabilities of the device were verified by identifying various brands of whiskey. By combining a small sensing module and a PDA, a considerably lightweight and portable electronic nose system was achieved.

Although typical electronic noses require a PC to acquire and process the signals from the sensor array, the PC, which was used as a pattern recognition engine, can be replaced with a powerful central microcontroller equipped with an embedded odor classification program [56,57,59–61]. This reduces the volume and weight of a digital apparatus used as an individual smart portable electronic nose device.

When designing this type of embedded electronic nose system, the requirements of computing power vary according to the complexity of the application. In particular, programming-embedded-pattern-recognition software that is suitable for the application is the key to appraising the computing power requirement. Similar to mammalian brains, a training or database-building procedure must be completed in the odor recognition algorithm, and these procedures are the most complex phase in the algorithm. Furthermore, the microprocessor should be evaluated according to the power consumption, system operation frequency, data capacity, instrument size limitation, manufacturing cost, and compatibility with other electronic devices, such as through Ethernet, buses, ports, and display interfaces. Table 2 shows the architectural options to the design of an embedded electronic system, from simple to complex system in form [56].

## 3. Chemiresistive Sensors

In electronic noses, the sensor array is the critical component of the system, and the designer must select a suitable type of sensor to build the array, such as conductivity sensors, mass piezoelectric sensors, optical sensors, or MOSFET sensors [82]. Depending on the specific features, the chemiresistive sensor, one of the conductive sensors, is highly applicable to compact electronic noses because of its simple electrical properties and readout interface circuit. The chemiresistive sensor can be divided into two types, namely, metal oxide and conductive polymer, and these two types of sensor display a variety of resistance values when exposed to odors [54]. Both types have the ability to form a sensor array, and these arrays consist of several sensors that exhibit differing sensitivity and selectivity. Several microsensors can also be integrated into a single small substrate, forming a miniature sensor array [83,84] that is combined with a resistive readout interface circuit [85]. Both metal oxide and conductive polymer sensors have unique advantages and disadvantages, as shown in Table 3. Noise behavior defines the detection limits of chemiresistive sensors and noise derives from external environmental interferences and inherent resistor noise. In DC resistance measurement, the flicker noise often determines the sensitivity threshold. After comprehensive consideration, the noise behavior restricts the sensitivity of the conductive polymer sensors to thresholds in the sub-ppm range [86]. However, the sensing film exhibits surface interactions, which is also a source to increase the noise level. The principle of noise behavior is the same for metal-oxide semiconductor gas sensors. The sensitivity of a metal-oxide sensor can be about ten parts per million (ppm) [87]. A severe disadvantage of metal oxides is that their operating temperature is typically very high, and the sensor must be able to sense at a temperature of about 300 °C [88]. Compared with metal oxides, the conductive polymer sensor can operate at an ambient temperature; therefore, there is no need for a heater. Moreover, the electronic interface of a conductive polymer sensor is simple; therefore, it is particularly suitable for portable instruments. The sensitivity of the sensor can also be in the range of 15 ppm. The main disadvantage of a conductive polymer sensor is that it is highly sensitive to humidity [89]; therefore, it is necessary to eliminate background humidity and control the sensor baseline drift when using a conductive polymer sensor.

### 3.1. Metal-Oxide Semiconductor Gas Sensor

Metal-oxide sensors have been commercially applied and widely used in electronic noses, as shown in Table 1. In 1984, a tin-oxide gas sensor (FIGARO^®^) was proposed for gas detection and applied in a gas-monitoring alarm [90]. Metal-oxide sensors have the advantage of strong sensitivity, and they can enable quick response to gas detection. In sensor arrays, gas selectivity can be increased by coating various noble metals (Ni, Pd, Pt, and Os) for each sensor [91,92]. As mentioned, the obvious drawback of metal-oxide sensors is that they operate at high temperatures, necessitating heaters and high power consumption. However, the gas sensitivity of the sensing film is also influenced by the quality of the heater that controls device temperature stability [93]. Emerging issues for enhancing performance and reducing size are discussed as follows. A microheater was designed and fabricated for high temperature, low power consumption, and strong thermal uniformity; it achieved a thermal efficiency of 36 °C/mW [94]. To fabricate a low-power polymeric microhotplate, several metal-oxide gas sensors and a microheater can be integrated on a silicon substrate [95]. A metal-oxide microsensor was designed for the selective detection of part per billion (ppb) levels to quickly monitor human-exhaled substances [96]. To enhance the sensitivity, zeolites were used to screen-print a layer of chromium titanium oxide [97]. A metal-oxide based oxygen gas sensor grew vertically aligned ZnO nanowires on ZnO:Ga/glass templates, and it featured the potential to operate at room temperature [98]. Another study analyzed the benefit of using a nanowire gas sensor to detect chemical warfare agents (CWAs), and its gas-detection capability was improved [99].

**Figure 3.**
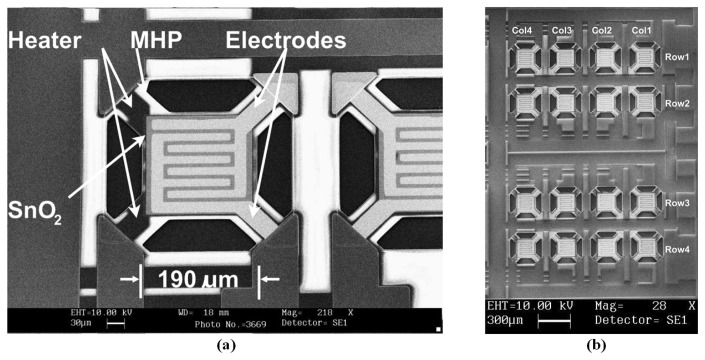
The SEM picture of the integrated tin oxide gas sensor array of (**a**) the single sensor element, and (**b**) the 4 × 4 gas sensor array. Reprinted with permission from [100].

In summary, chemiresistive metal-oxide nanostructures, such as nanowires, nanotubes, and nanofibers, have attracted attention regarding their application in miniature electronic noses in the last decade [101,102]. To benefit the advance of fabrication technology, metal-oxide sensors can be integrated in a single microsensor chip for electronic nose systems [103], and they could be improved in both the size and overall power consumption [104]. For example, a microsensor gas array based on a metal-oxide structure was integrated with 12 tin-oxide sensing elements and a single microheater in one substrate for odor detection. A single sensor was reduced to a minimal area of 10 × 30 μm^2^, and the device size was set to 8 × 8 mm. The fair gas sensitivity was maintained at 290–300 °C, and the heating power was approximately 50 mW at 300 °C [105]. A convex microhotplate structure of surface micromachining technology was applied to fabricate an integrated 4 × 4 tin-oxide gas sensor array [100], as shown in Figure 3. The gas sensor array occupied an area of 2 × 2.8 mm^2^, and the sensor pitch was 370 μm. The microhotplate was 190 × 190 μm^2^ and exhibited a 2.8 μm polysilicon sacrificial layer. It exhibited the advantages of a simple process procedure and CMOS compatibility, a maximal curvature of 2.438 cm^−1^, and a maximal thermal efficiency of 13 °C/mW. Furthermore, the selectivity of the tin-oxide sensor array could be modified using metal additives and ion implantations [106]. Certain research has emerged on this type of microsensor array, which can be applied in a clinical diagnostics as a temperature controller for noninvasively detecting disease biomarkers [107]. To enhance the gas selectivity, the design of microarrays for electronic nose instruments was employed by electric potential over a MOX film [108].

The performance of the one-chip array is also critical for gas identification. Based on the aforementioned research on microhotplate-based SnO_2_ thin-film sensors, the on-chip array could be combined with and analyzed using a back-end pattern recognition engine for gas identification. In this case, the sensing signals could be analyzed using the combined system of the five classifiers, multilayer perceptron (MLP), Gaussian mixture models (GMM), radial basis function (RBF), K-nearest neighbors (KNN), and probabilistic principal component analysis (PPCA), and the individual classifiers [109]. For odor discrimination, a thick-film tin-oxide sensor array was fabricated and employed in a neural network algorithm for analysis and classification [110]. This also means that a portable electronic nose based on a micro-resistive sensor array could be easy to implement and verify. In [111], a portable electronic nose based on a sensor array equipped with a polysilicon heater was analyzed using principal component analysis (PCA) plots for the performance of the sensor array and exhibited 100% accuracy in a probabilistic neural network (PNN). However, it is difficult to integrate numerous metal-oxide sensors on a single small substrate, and power consumption remains a limiting factor. The number of metal-oxide sensors required for electronic noses is typically numbering between several and several tens, but metal-oxide sensors remain the most favorable optimal choice for miniature and integrated electronic noses.

### 3.2. Conductive-Polymer Gas Sensor

The chemiresistive gas sensor based on conduction polymers uses intrinsically conductive polymers as the sensing active layer. Such polymer-based sensors are used for chemical vapor sensing. After exposure to chemical vapors, the active sensing materials interact with the chemical vapors, and the doping level in conductive polymers transfers electrons to or from the analytes, causing conductivity changes [112,113]. Incorporating a second component, such as insulating polymers, into the conductive polymer film is one of the crucial methods of developing original sensors [39,114]. Unlike modifying the structure of a conductive polymer, these composite materials can avoid the need for complicated chemical syntheses processing. Unlike metal-oxide gas sensors, conductive polymer gas sensor can operate at an ambient temperature; because there is no need for a heater, conductive polymer gas sensors exhibit a considerably lower power consumption. Moreover, the electronic interface of conductive polymer sensors is simple. Moreover, the size of the interface could be reduced by using application-specific IC (ASIC), and an ASIC based on a current-mode multiplexer has been proposed for connecting with 32 conductive polymer gas sensors for portable electronic nose applications. The electronic interface of conductive polymer sensors is simple; thus, it is particularly suitable for portable instruments [115]. For electronic noses, the sensor array consists of various sensor elements, which are coated with various types of synthesized conductive polymer materials; therefore, the sensors could exhibit different sensitivity and selectivity [116]. Because of the simple structure of the device, small conductive polymer gas sensors can be fabricated easily. In 1991, an integrated gas sensor was proposed that the sensing material could be deposited on a simple four-finger electrode, and the electrode was a gold-plated 13 mm^2^ alumina tile 0.6 mm in thickness [117]. A conductive polymer gas sensor was fabricated using a CMOS-compatible process, forming a silicon micro-bridge composed of four resistive elements. A precision analogue interface circuit was used to display information [118].

The conductive polymer has difficulty of generating a variety of sensors, causing a disadvantage when forming a sensor array. Therefore, a subset of conductive polymer gas sensor technology, the conductive polymer composite sensor, is used, which is fabricated by coating or encapsulating a mix of conductive and non-conductive materials on an electrode surface. The polymer is the non-conductive material of a specific receptor agent; it can absorb and desorb the target in the vapor in the early and late vapor-diffusion stages. The conductive materials contribute electrical conductivity to the sensing films and the polymers swell to increase the resistance level when exposed to a vapor [119–121]. In conductive polymer gas sensors, various polymers are sensitive to water vapor [122,123] and dependent on the temperature [124,125]. The main disadvantage of a conductive polymer sensor, particularly in the conductive polymer composite sensor, is that it is highly sensitive to humidity; therefore, it is necessary to eliminate background humidity and control the sensor baseline drift when using a conductive polymer sensor. The vapor pressure also affects the response of the sensing film, but this effect is substantially uncommon in open spaces [126]. To enhance the sensing ability, the materials were diversely modified. The metal can be a conductive material, generating a rapid change in resistance (>7 decades) in the vapor sensing phase [127]. Compared with traditional silicone rubber or carbon-black material, the silicone rubber/graphite composite material exhibits a superior vapor-sensitive response because of their porous structures [128]. In previous research, silicone rubber/acetylene black films [129] and reactive hydroxyl-terminated polybutadiene liquid rubber/carbon black conductive films [130] were applied in vapor sensing. Adding the tiny conducting materials into the polymers is a method of enhancing the response of sensing films; carbon aerogels have also been applied to fill polystyrene and improve these responsivity and adsorption behaviors [131]. Filling multi-walled carbon nanotubes in polystyrene enhanced the sensitivity for the mixing vapors [132]. A conductive composite fabricated by filling polystyrene with hybrid fillers composed of vapor-grown carbon nanofibers and carbon black exhibited strong vapor sensitivity because of the formation of specific conductive pathways in the matrix [133]. The sensing material based on a vapor-grown carbon fiber surface and grafted branched polymers exhibited the ability to suddenly increase and decrease resistance in the vapor absorption and desorption phases. [134]. A highly selective sensing film has been proposed, which was attributed to the properties of multi-walled carbon nanotubes that contained carboxyl groups grafted to poly(ethylene glycol) polymers [135]. The film, based on an expanded graphite/poly(methyl acrylic acid) composite, increased the resistance change ratio for vapor sensing, contrasting with the natural flake graphite/poly(methyl acrylic acid) composites [136]. Using compatible polymer blends to fabricate a carbon black-polymer composite for various sensor types has been proposed to detect vapors [137]. After changing binders contained in SWNTs/silane sol solution, the selectivity of carbon-nanotube (CNT)-based vapor sensors improved to exhibit well sensitivity for alcohol vapor [138].

**Figure 4.**
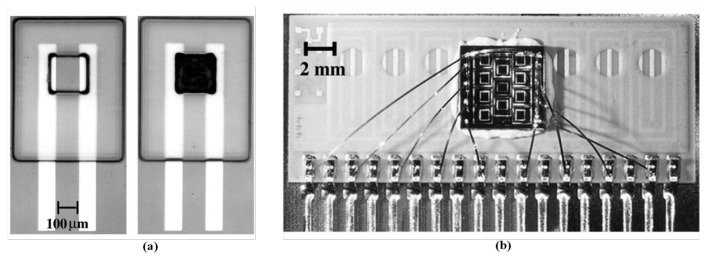
(**a**) The well-defined region for depositing sensing material, occupying an area of 100 × 100 μm^2^. The photograph shows the sensors before and after deposition. (**b**) The gas sensor array had been integrated into one chip. Reprinted with permission from [139].

A carbon-black (CB)-based miniature gas sensor array as shown in Figure 4 was fabricated on a small silicon substrate. Six polymer CB composite films were deposited between two lead electrodes, forming the sensing film, and the deposition region was defined by the “well” of the SU-8. The device was fabricated at a size of approximately 0.30 mm^2^, and the minimal sensing area was approximately 100 × 100 μm^2^. The sensitivity of the small sensors was between 2,000 and 10,000 ppm, and the device featured a linear response for organic vapors. This sensor array was designed to enable integration with resistive readout circuits [139]. Although miniaturized conductive polymer gas sensors have the advantage of operating at an ambient temperature, sensing characteristics could be improved by integrating a stable heater on the silicon substrate. The stability, ability of reaction, and baseline level are all related to the operation temperature. The sensor device was integrated with an interdigitated electrode pair, a microheater, and a micro-machined well exhibiting an area of 2 × 2 mm^2^, and subsequently employed in a sensor array consisting of 16 separate sensors on one 30 × 14 mm^2^ chip. The temperature-controlled Pt microheater consumed only 7 mW to heat the film at the maintained temperature of 40 °C in the sensing period [140,141]. Based on the result, it was applied in the portable electronic nose system discussed in Section 2 [81]. The results of composite material sensor coating also determine the sensor reproducibility. A uniform thin film is crucial for microsensors with small active layers, allowing the film to contribute to efficient field-effect mobility and reduce noise [86]. Various techniques can be used to coat the chemiresistive thin film, including screen printing [142], spin coating [143], spraying [144], ink-jet printing [145], and imprinting [146]. To solve the poor reproducibility, the biomimetic two-layer multiple-walled carbon nanotube (MWNT)–polymer composite sensor was presented to overcome the problem, and its fabrication procedure was simple. The quality of the sensing film was easily controlled, maintaining the well sensitivity and stability. The sensor array was applied in a portable electronic nose to identify complex samples, such as sake, sorghum liquor, medical liquor, and whisky [147]. In addition, the power of a sensor array can be quantized to evaluate the performance levels of sensing tasks [148], and the PCA+KNN algorithm has also been proposed to estimate the ability of the sensor array to identify odor [149].

**Figure 5.**
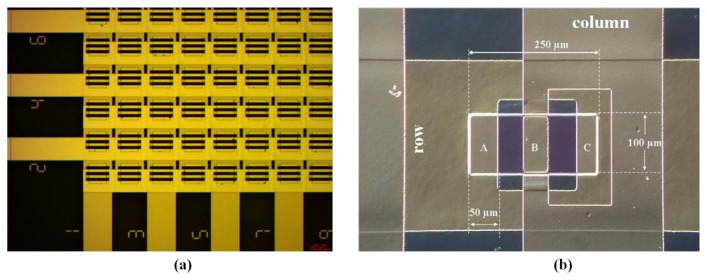
(**a**) The photo shows one corner of the sensor array. The element has the dimension of 220 × 220 μm^2^ with 20 μm gap between the electrodes. (**b**) Single transducer element, the sensing material would be deposited to cover A–C. Reprint the photo in [151].

In a biomimetic artificial olfaction system, a sensor array based on conductive polymer exhibits clear superiority and potential. In mammalian nasal cavities, there are 6–10 million olfactory receptors comprising 900 different types. Therefore, the sensitivity and selectivity of the artificial olfactory system could be improved by increasing the numbers and types of sensor. Therefore, a chemiresistive microsensor array was developed to integrate 80 elements in a 10 × 10 mm^2^ silicon die, and it was coated with a CB-polymer composite film [150]. In an advanced European project, NEUROCHEM, a conductive-polymer-based sensor array was composed of 16,384 elements spread across four smaller arrays of 64 × 64 interdigitated electrodes on a borosilicate substrate, as shown in Figure 5. The project target was to build an array comprising 216 (= 65,536) elements for a biomimetic olfaction system. A prototype that could read out the sensor signals from a 65,536-sensing-element array was developed [151,152]. Because of the large number of sensors, the method and interface for sensor readout was a critical issue; this is discussed further in Section 4.3.

## 4. Interface of the Chemiresistive Sensors

The purpose of a chemiresistive sensor interface is to quantify and display the resistive values of sensors and then convert them to a signal that could be processed by the pattern recognition engine. Therefore, the interface plays a critical role between sensors and the pattern recognition engine in the electronic nose system. Furthermore, certain systems have other advanced features, such as eliminating the initial baseline shift of the sensor resistance caused by the environment and controlling the temperature of the metal-oxide sensor heater. Because of the progression of CMOS and MEMS technology, sensor interfaces can be fabricated through CMOS and MEMS processing. The design consideration to modify the sensor interfaces was thoroughly discussed in [153], and interfaces for gas sensors were also comprehensively reviewed in [154]. To conduct a comprehensive survey of the interface, the resistive sensor readout circuit can be divided into two basic categories: (1) in an analog-to-digital converter (ADC) based interface, the resistive information is converted from the sensor to an analog voltage signal, and then converted to a digital signal through an ADC. (2) In a pulse width modulator (PWM) based interface, the value of resistor is converted to the width of pulse. The different pulse widths represent different resistor values, and can be directly calculated using a digital unit such as counter. Both ADC-based and PWM-based interfaces have been proposed for application in chemiresistive electronic nose systems. In summary, chemiresistive sensors and chemiresistive sensor-based electronic nose systems contribute three main advantages: (1) potential for mass production and reliable manufacturing to reduce cost; (2) few discrete components, which reduces the volume, weight, and power consumption; and (3) the capability of integrating MOS-compatible chemiresistive sensors and additional functional circuits. Because of the advantages, Hatfield used CMOS technology to fabricate an array interface for a chemiresistive electronic nose in 1994 [62]. The resistance of metal-oxide semiconductor gas sensors and conductive polymer gas sensors can be converted by different types of interface circuits. According to the type of interface, Section 4.1 presents ADC-based interfaces; Section 4.2 presents PWM-based interfaces; Section 4.3 presents interface circuits for large amounts of sensors. Additionally, CMOS technology used in integrating the sensor array and its interface circuit, as well as its application in integrated chemiresistive electronic noses, is emphasized.

### 4.1. ADC-Based Sensor Interface

**Figure 6.**
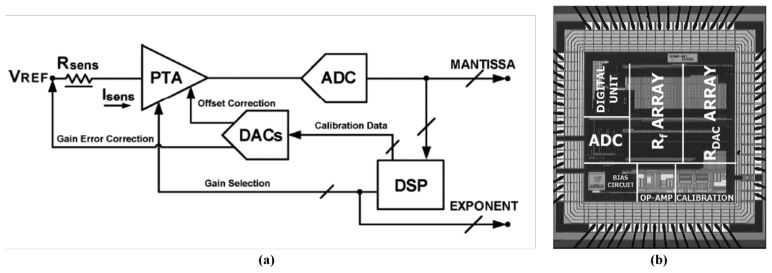
(**a**) Block diagram of the wide-dynamic-range resistive interface ASIC, and (**b**) the die photo of the 4-channel interface circuit ASIC. Reprinted with permission from [159].

The ADC-based sensor interface was developed to convert analog signal from sensor signal conditioning circuits to digital signal [62], and certain typical signal conditioning circuits for generating voltage signals were introduced and analyzed, such as a potential divider, an inverting amplifier, and a constant current source [155]. Based on these typical structures, two interface ASICs were proposed as the bridge between 32 conductive polymer gas sensors and a PC in a handheld electronic nose system [156]. The precise, wide-ranging resistive interface was designed to detect small changes for low concentration gas and eliminate the effect of baseline drift caused by background noise [157]. For the detection of ppb gas concentrations, a novel interface architecture was presented that primarily consisted of a fully-analog lock-in amplifier and an automatic phase alignment [158]. A wide-dynamic-range resistive interface ASIC as shown in Figure 6 was fabricated using 0.35 μm CMOS technology, occupying 3.1 mm^2^ and consuming 6 mW at a 3.3 V supply voltage. The interface was composed of a single-ended continuous-time programmable transresistance amplifier (PTA) and a 13-bit incremental ADC. Two 8-bit digital-to-analog converters (DACs) and a digital signal processing (DSP) unit were used in the feedback loop to automatically refine the PTA features matching sensor specifications. It achieved a high accuracy of over 0.1% and a particularly wide sensor resistance range (between 100Ω and 20 MΩ) [159]. The resistive interface ASIC was combined with a temperature-controlled heater chip to achieve a smart ADC front end, and the front end could be applied in a metal-oxide sensor array for portable integrated gas sensing applications, such as a portable integrated electronic nose [160]. The other challenges were to adapt the driving current of each sensor using an external CPU and read the double-ended voltage of the sensor. In other similar architecture, a sensor driver controlled by an external CPU adjusted the driving current of each sensor, and an interface subsequently readout the double-ended voltage of the sensor. This device also exhibited a wide sensor resistance range (from 500Ω to 1 MΩ) [161]. Other researchers designed a baseline cancellation circuit to read the changed resistor message efficiently. The circuit could eliminate the portion that was occupied by the baseline resistor of the sensor by using the full ADC resolution [162]. In summary, the ADC-based sensor interface is the basic structure type for both metal-oxide semiconductor gas sensors and conductive polymer gas sensors. According to the degree of system resolution requirement, the interface can vary from a simple structure to a complex structure. Because of the baseline drift problem, chemiresistive gas sensor requires a baseline cancellation interface circuit. Regarding oxide semiconductor gas sensors, a smart heater controlling circuit is required, and the circuit can be implemented by using system feedback.

### 4.2. PWM-Based Sensor Interface

**Figure 7.**
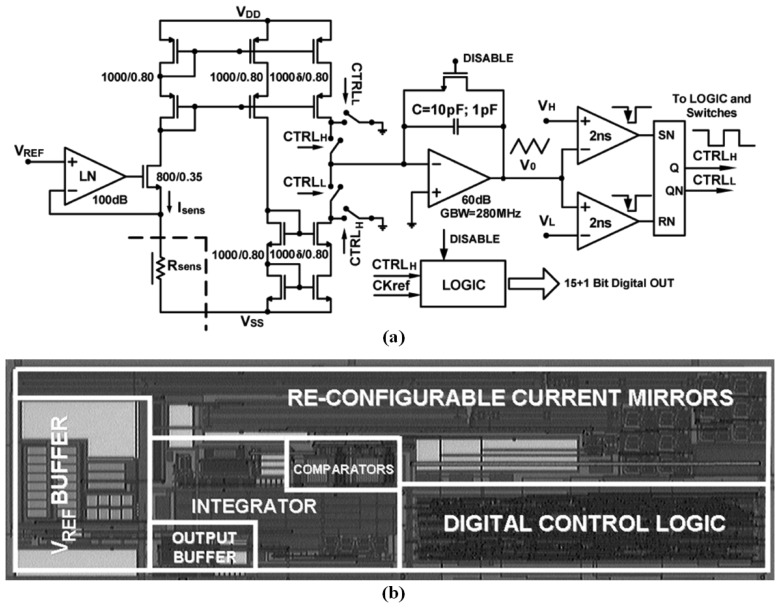
The PWM-based interface circuit: (**a**) schematic, and (**b**) photograph of silicon prototype. Reprinted with permission from [164].

The operation principle of the PWM-based sensor interface is the delay of resistor–capacitor (RC) charge and discharge. When the chemiresistive sensor generates a resistor change, the RC charge/discharge time differed, implying a different pulse width. Pulse widths were able to be detected, and could be converted back to the sensor resistances. This PWM signal can be easily obtained using digital units, such as a multipoint control unit (MCU), so that an interface is not required for further ADCs or other analog components. A PWM-based interface ASIC consisted of a ring-oscillator formed by a chain of three inverter stages and an RC delay stage formed by a chemiresistive sensor and an external capacitor. Thus, the output frequency of the oscillator corresponded with the resistance value of the sensor [163]. A wide-dynamic-range interface circuit as shown in Figure 7 was based on another resistance-to-frequency conversion, and the sensor resistor determined the charged and discharged currents, thus dominating the frequency. The interface was implemented using 0.35 μm CMOS technology, exhibiting a precision of 0.4%, a sensing resistive range from 1 kΩ to 1 GΩ, and costing 15 mW at a supply voltage of 3.3 V [164]; the interface was further improved to a wider detection rage of 100 KΩ to over 100 GΩ [165], and an ASIC was implemented [166]. A prototype based on a resistance-to-period converter was realized as a measurement platform that connected to eight metal-oxide sensors. Its relative displacement with respect to the reference line was less than 1%, between 10 kΩ and 3 GΩ [74]. A similar design was proposed to approach a PWM-based interface ASIC that operated at a low ±1.0 V supply voltage and costed only 780 μW for each channel [167]. This interface was developed for a low-cost electronic nose [168]. The Second Generation Current Conveyor (CCII)-based interface was designed to operate at a low ±0.75 V supply voltage and cost only 700 μW [169]. In previous studies, a fast readout interface was designed [170], and a CMOS-integrated interface was fabricated using 0.35 μm standard CMOS technology, consumed only 600 μW at a supply voltage of 1.8 V, and cost approximately 0.9 mm^2^ [171].

A metal-oxide sensor requires a heater for temperature control. Improving the selectivity, sensitivity, and stability of an interface module including a readout interface and heater management was discussed in [172]. For a metal-oxide sensor, the PWM-based interface offers further advantages: it is easy to combine with a temperature-controlling circuit heater. An ASIC, fabricated using AMI Semiconductor (AMIS) 0.7 μm CMOS technology, consisted of temperature-controlled and PWM-based interface circuits, and exhibited an operating temperature of 100 to 425 °C and a sensing resistive range of 50 kΩ to 3.3 MΩ [173]. A programmable PWM interface offering a variable duty cycle signal was applied to control the power of the heating resistor; its range in the electronic nose system was 100–300Ω [174]. In short, the PWM-based interface could reduce the effect of ADC; this PWM signal style is particularly convenient for use in the heater controlling circuits of metal-oxide semiconductor gas sensors.

### 4.3. Large Amounts of Sensor Interfaces

**Figure 8.**
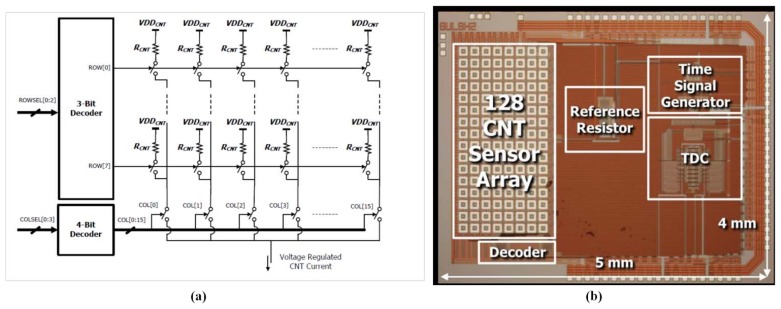
The row–column interface integrated with 128 SnO_2_-CNT gas sensors, (**a**) block diagram, and (**b**) chip photograph. Reprinted with permission from [175].

In recent years, biomimetic olfactory systems have attracted an increasing amount of attention; one of the features of the system is the requirement for large amounts of sensors to mimic mammalian receptors. Systems featuring large amounts of sensors (typically in the hundreds) have been designed using mature gas sensor integration and manufacturing technology [150–152]. Because of the growth in the number of sensors, the hardware cost of the interface increases rapidly; traditionally, an N × M resistive sensor array required N × M interface channels to read out the sensors. A row–column readout structure was presented to simplify the complexity of traditional N × M resistive interfaces to the size of N + M [176]. This design was conceived to minimize the effect of the crosstalk between the interconnection lines and various elements. Following the interface structure, a biomimetic olfaction system was designed based on an electronic readout prototype board with a size of 16 × 10 cm^2^ for a 64 × 64 resistive gas sensor array, and the prototype board was controlled by an field-programmable gate array (FPGA) [151]. To realize a biomimetic olfaction system with 16,384 sensing elements, the system was designed using four sensor chips (each chip included 64 × 64 interdigitated electrodes), electronic readout prototype boards, and a data transmission card connected to a PC [152]. Thus, the row–column structure combined with the PWM-based interface was presented to readout a sensor array, which contained 128 SnO_2_-CNT gas sensory cells, as shown in Figure 8. In addition, the interface was integrated with the sensors in one chip, and it was fabricated using a 0.35 μm CMOS process, occupying 5 × 4 mm^2^ of chip area, and consumed only 30 mA at a 5 V supply voltage [175]. Integrated technology in the sensors and interface is discussed further in Section 5.1. Compared with metal-oxide semiconductor gas sensors that require operation at high temperatures, conductive polymer gas sensors can operate at ambient temperatures, drastically reducing power consumption. Therefore, biomimetic olfactory systems tend to adopt conductive polymers to form the sensor array and therefore numerous sensor interfaces are designed for the sensor array.

## 5. Highly Integrated ASIC/SoC for Electronic Nose

Integrated microsystems, connected with the sensing modules, have been merged with data acquisition circuits and computing elements in a single chip to form a small and compact device [177]. The sensing modules were also fabricated using several types of microsensors and their microelectronic functional blocks on a single chip [178,179]. The results imply that the proposed system could also consist of a chemiresistive sensor array, interface circuits, and a pattern recognition engine in a single chip, or in multi-chips to achieve a highly integrated, low power, and small electronic nose device. This chapter presents several emerging, highly integrated technologies that could be applied in an electronic nose system-on-chip (SoC). According to the applications of highly integrated chip, Section 5.1 presents a sensing frontend ASIC, and Section 5.2 presents a very large scale integration of artificial neural networks for electronic nose.

### 5.1. Sensing Front End ASIC and Electronic Nose SoC

**Figure 9.**
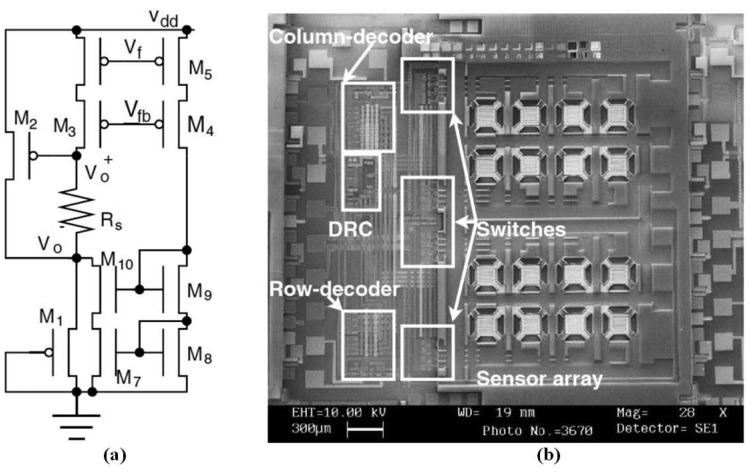
(**a**) The differential sensor conditioning circuitry of the read-out circuit and (**b**) the photo of the integration of SnO2 gas sensors and its differential preprocessing circuits in one chip. Reprinted with permission from [183].

**Figure 10.**
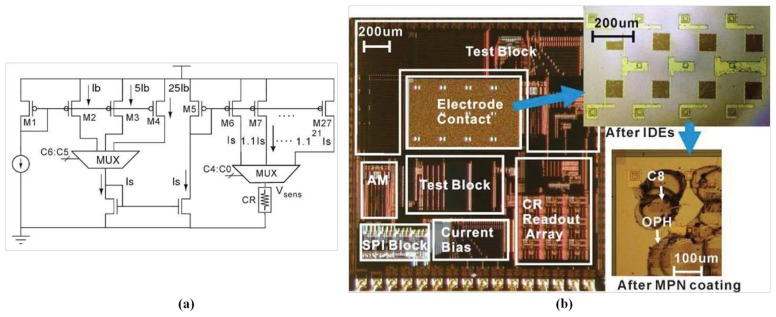
(**a**) A wide-range programmable sensor conditioning circuitry and (**b**) the die photo of the CR-array and its readout circuit. Reprinted with permission from [184].

For gas sensing, most researchers have focused on the integration of chemiresistive sensor arrays, temperature-controlled circuits, and the readout interface to form a sensing front end of the electronic nose. The IC technology can be applied to implement the electronic nose system and also to integrate parts of the system into one chip. An ASIC has been designed to monitor volatile organic compounds (VOCs), and was integrated with two polymeric chemiresistors and smart interface circuitry in a single chip through a standard Alcatel Microelectronics 0.7 μm CMOS process, occupying a chip area of 3,300 × 3,750 μm^2^. The ASIC can be controlled using an off-chip micro-controller to form a self-calibrating, programmable, palm-top gas detecting device [180]. The ASIC contained a SnO_2_ CMOS sensor and its temperature-controlled digital circuits, costing the heated area 300 × 300 μm^2^ and achieving a maximum of 400 °C at a supply voltage of 5.5 V [181]. Additional, sensors were integrated into the ASIC, which was occupied a 5,500 × 4,500 μm^2^ chip area and was fabricated through a 0.6 μm 2-P 3-M CMOS process [182]. Based on the integrated 4 × 4 SnO_2_ oxide gas sensor array, mentioned in [105], it was integrated with row–column multiplexing and differential read-out circuitry (DRC) through an in-house 5 μm process, as shown in Figure 9. The DRC allowed a constant current to flow through the sensor, causing a voltage drop between the two electrodes of the sensor, and then connected to a united-gain amplifier, generating a differential output at a 1/2 common-mode supply voltage [183]. Based on the baseline tracking and eliminating interface [162], a miniaturized chemiresistive gas sensor array, integrated with an 8-channel readout circuit, was fabricated to reduce the size of the device for analyzing complex mixed vapors, as shown in Figure 10. The chemiresistive arrays were coated with thiolate-monolayer-protected gold nanoparticle (MPN) films, and the interface featured a high resolution and a wide dynamic range. The wide-range interface controlled using system feedback offered a programmable exponential current bias flowing through the chemiresistor, according to the initial baseline resistor. This integrated chip, in which the 8-channel readout circuit occupied an area of 2,200 × 2,200 μm^2^, was fabricated through a 0.5 μm CMOS process and consumed only 66 μW of each channel at a supply voltage of 3.3 V [184].

**Figure 11.**
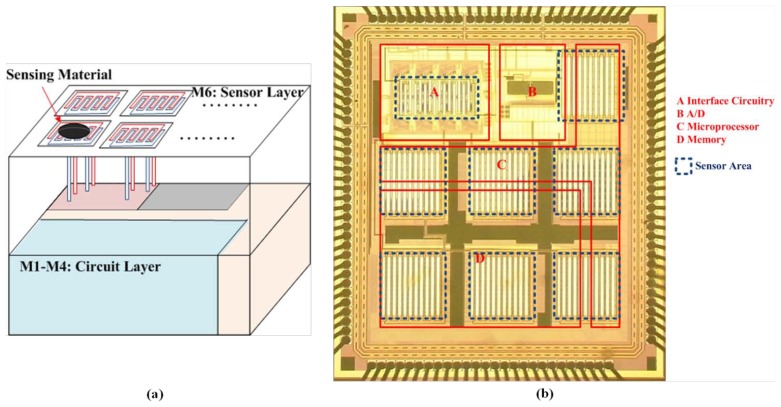
The electronic nose SoC (**a**) the concept of 3D structure and (**b**) die photo before coating the sensing materials. Reprinted with permission from [186].

In addition, another study designed an electronic nose signal-processing chip to efficiently process sensor signals in a portable and wearable electronic nose. The signal-processing chip could be connected directly to the sensor array, and different sensors could be selected according to the application. An analog electronic nose signal processing chip was proposed and consisted of four stages: a sensor stage (interface), a signal processing stage, a classifier stage, and a database stage. The interface was connected to the CB-polymer, generating 3D odor data, and the odor data was then normalized using a signal processing unit. Finally, this normalized data could be stored in a static random-access memory (SRAM) and used to calculate the Euclidean distance for odor classification. This analog electronic nose signal-processing chip was fabricated through an AMI_ABN 1.5 μm double-poly double-metal process. The chip occupied an area of 2,117 × 2,117 μm^2^, cost 7.6 mW when exhibited 100% resistance change, and 1.3 mW without gas detection in standby mode [185]. For more powerful and flexible processing capabilities, the analog circuits of the signal processing stage and classifier stage could be replaced by a simple microprocessor, as proposed in [57]. A previous study proposed an electronic nose signal-processing chip consisting of interface circuitry, an ADC, memory, and a microprocessor embedded with a KNN recognition algorithm. It was connected to an MWNT-polymer sensor array chip [147] to form a portable electronic nose device. The chip was fabricated through a Taiwan Semiconductor Manufacturing Company (TSMC) 0.18 μm 1P6M CMOS technology process and occupied an area of 2,058 × 1,952 μm^2^, consuming 2.81 mW at a supply voltage of 1.8 V [65]. Furthermore, based on the electronic nose signal-processing chip [65], a fully integrated electronic nose SoC design allowed the fabrication of a lightweight, low-power-consumption, and wearable electronic nose chip. In addition to the basic architecture of the electronic nose signal-processing chip, the SoC design also contained eight on-chip integrated sensors, and a post-MEMs process was not performed. In the SoC design, interdigitated electrodes were implemented with top metal layer (metal 6) to form a 3D structure, and the interdigitated electrodes were connected to the interface circuits by the lower metal layer (metal 1 to metal 5). The sensor area could be defined by the mask to remove the surface protection. After removing the surface protection, the sensing materials could be deposited on the interdigitated electrodes, forming an on-chip sensor array [186] as shown in Figure 11.

In summary, metal-oxide semiconductor gas sensors require specific CMOS or MEMS processing. The complexity of circuits is restricted such that the metal-oxide semiconductor gas sensors are suitable for integration with simple backend CMOS circuits for ASICs. In contrast to metal-oxide semiconductor gas sensors, conductive polymer gas sensors can be implemented by coating the materials on two simple electrodes, and operated at ambient temperatures, thereby drastically reducing power consumption. Because of these benefits, conductive polymer gas sensors are suitable for implementation as electronic nose SoCs. However, a conductive polymer based electronic nose SoC must also address the non-ideal fabrication factors such as fabrication variation and reproducibility.

### 5.2. Very Large Scale Integration of Artificial Neural Networks

**Figure 12.**
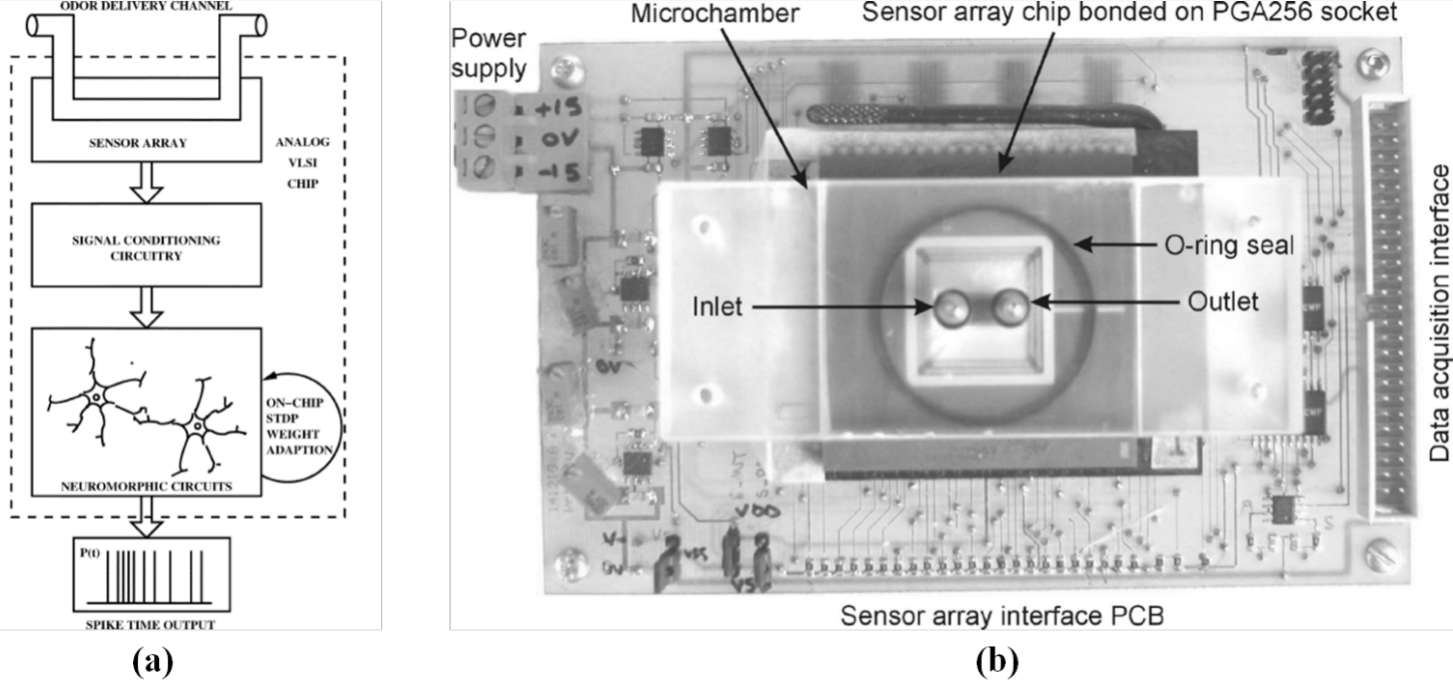
(**a**) Adaptive neuromorphic olfaction system. (**b**) The top view of the olfaction chip and chemosensor array, mounting on top microchamber. Reprinted with permission from [191].

Artificial neural networks (ANN) have been employed in electronic noses as pattern recognition engines, and have offered the ability of odor identification in various areas [187,188]. Because of the computational complexity, ANN algorithm requires a long execution time and a powerful computer or microprocessor and is therefore not suitable for a portable, low-cost electronic nose device. Thus, the very-large-scale integration (VLSI) implementation of an artificial neural network offers the advantage of low power and high parallel-processing capability. An analog ASIC comprises a multilayer perceptron neural network (MLPNN), which has been proposed as a low-power and small-area analog classifier for electronic noses. The ASIC consists of four input neurons, four hidden neurons, and one output neuron, and was fabricated through a TSMC 0.18 μm standard CMOS process. This circuit consumes 0.553 mW at a voltage supply of 1.8 V and occupies an area of 1,360 × 1,360 μm^2^. This MLPNN ASIC has been tested for its capability to process and identify three types of fruit odors, and achieved an accuracy of 91.7% [189]. A biomimetic spiking neural network (SNN) ASIC was used in producing a low-power, small odor classifier for electronic noses. The features of sub-threshold oscillation and onset-latency representation were used to enlarge the distance of odor distribution between each type. In this structure, the synaptic weights converged according to the spike-timing-dependent plasticity learning rule between the mitral and cortical cells. This SNN ASIC was implemented through a TSMC 0.18 μm 1P6M standard CMOS technology, occupied an area of 1.78 mm^2^, consumed 3.6 μW at a supply voltage of 1 V, and achieved 87.59% accuracy for classifying odor data [63]. A CMOS gas recognition chip for encoding the output of a metal-oxide sensor array [100] into 2D spatio-temporal spike signatures was presented. The sequential spike signatures were drift insensitive and concentration invariant, and the features of the odor signal were maintained. The chip was fabricated through a 0.35 μm CMOS process, occupied an area of 1,550 × 1,710 μm^2^, cost a power consumption of 6.6 mW, and achieved a detection rate of 94.9% [190]. An adaptive neuromorphic olfaction system, as shown in Figure 12, consisting of several silicon chips was fabricated through an Austria Micro Systems (AMS, Styria, Austria) 0.6 μm CMOS process. This system featured an chemiresistive sensor array coated with a CB-polymer sensing film, an dc cancellation interface to cancel the baseline sensor, and an adaptive neuromorphic circuit realizing neurons and synapses based on two operational transconductance amplifier and capacitor (OTA-C) structures and spike-timing-dependent plasticity learning (STDP) circuits. This work also intended to implement a fully integrated olfaction system, but mentioned several challenges, such as the long term analog weight storage problem, the mismatch caused by the fabrication variation, and the poor reproducibility of the chemiresistive sensor array [191].

## 4. Conclusions

This study presented a review of the research activities regarding chemiresistive sensors integrated in an electronic nose. Because of the advances in CMOS/MEMS technology, electronic nose systems consisting of sensor arrays, electronic interfaces, and pattern recognition engines could achieve a maximal degree of integration, enabling a small, fast, and inexpensive electronic chip to be realized. According to different applications, the structure of the embedded electronic nose systems could be simple or complex. Two chemiresistive gas sensors, metal-oxide semiconductor and conductive polymer, were introduced and are suitable for integration in sensor arrays in a single small chip. The resistive electronic interface based on the PWM and ADC was also discussed, and the row-column interface for large amounts of sensors for biomimetic olfaction systems was addressed. Finally, highly integrated ASIC and SoC designs for electronic noses were mentioned, such as the sensing front-end chip design, which combines the sensor array and its readout interface, the electronic nose signal-processing chip design, which combines the specific interfaces and hardware of pattern recognition engines and electronic nose SoC designs, which allow a fully integrated electronic nose system to be fabricated on a single chip. In summary, the chemiresistive gas sensor electronic nose has the opportunity and potential to achieve the target of becoming a $1 device [51]. It could therefore become an indispensable device in daily life.

## Figures and Tables

**Table 1.** Commercial and available electronic nose instruments, modified from [52–54].

**Manufacturer**	**Model**	**Sensor Array Type/Technology**	**Size**
Agilent, http://www.chem.agilent.com/	4440A	Fingerprint of MS	Desktop
AIRSENSE Analytics, http://www.airsense.com/	i-PEN/MOD PEN3	MOX MOX	Laptop Laptop
Alpha MOS, http://www.alpha-mos.com/	FOX 2000, 3000, 4000 Gemini Heracles AIRSENSE	MOX MOX Ultra Fast GC with two column Soft IMS	Desktop Desktop Desktop Desktop
AltraSens, http://www.altrasens.de/	OdourVector	QCM	Desktop
AppliedSensor, http://www.appliedsensor.com/	Air Quality Module	MOX	Laptop
Aromascan PLC, http://www.aromascan.com/	A32S	CP	Desktop
Dr. Foedisch AG, http://www.foedisch.de/	OMD 98	MOX	Laptop
Draeger, http://www.draeger-safety.com/	Multi-IMS MSI 150 Pro2i	IMS MOX	Palmtop Laptop
Electronic Sensor Technology, http://www.estcal.com/	ZNose 4200, 4300, 4600 ZNose 7100	GC and SAW GC and SAW	Laptop Laptop
Environics, http://www.environics.fi/	M90-D1-C ChemPro100	IMS IMS	Laptop Palmtop
Forschungszentrum Karlsruhe, http://www.fzk.de/	SAGAS	SAW	Laptop
GSG Mess- und Analysengeräte, http://www.gsg-analytical.com/	MOSES II	Modular Gas Sensor Array: QCM, MOX	Laptop
Owlstone Nanotech, Inc., http://www.owlstonenanotech.com/	Lonestar	IMS	Laptop
Rae Systems, http://www.raesystems.com/	ChemRAE	IMS	Palmtop
RST-Rostock, http://www.rst-rostock.de/	FF2, FF2D GFD1	MOX MOX	Desktop Desktop
Sacmi, http://www.sacmi.eu/	EOS Ambiente	MOX	Desktop
SMart Nose, http://smartnose.com/	SMart Nose 2000	Fingerprint of MS	Desktop
Smith Group, http://www.smithsdetection.com/	Cyranose 320 GID-2A, 3 SABRE 4000 ADP 2000	CP IMS IMS IMS	Palmtop Desktop Desktop Palmtop
Sysca AG, http://www.sysca-ag.de/	Artinose	MOX	N/A

CP, conductive polymer; MOX, metal-oxide semiconductor; IR, infra red; SAW, surface acoustic wave; QCM, quartz crystal microbalance; QMS, quadrupole mass spectrometry; GC, gas chromatography; IMS, ion mobility spectrometry.

**Table 2.** Architectural alternatives in the design of an electronic nose. Reprinted with permission from [56].

**Architecture**	**Typical Configuration (bit/speed/RAM)**	**Pros/Cons**	**Typical Programming Language**	**Available Processing**
Sensor Array + μC (PIC)	8 bit/10 MHz/k bytes	Easy, small, low power, portable, cheaper	ASM/C	Easy algorithms with few data, KNN, easy NN, mostly trained off-system, linear classifiers, quadratic classifiers.
SA + high perf. MC	8–16 bit/ 10–33 MHz/k bytes	Small, low power, portable, cheap	ASM/C	Some small matrix manipulation available, linear (PCA, LDA, PCR), KNN, easy fuzzy interface Systems.
SA +μP or DSP	16–32 bit/ 20–100 MHz/Mb	Very fast, medium size, portable, high power consumption	ASM/C/C++	Linear (PCA, LDA, PCR, PLS), KNN, easy neural and fuzzy system, standard feature extraction/selection (PCA, LDA).
SA + Embedded PC	32 bit/ 80–233 MHz/Mb	Fast, medium size, portable, huge data capacity, high consume expensive	Any	Linear, complex learning algorithms (GA, NeuroFuzzy Systems, mixture models, APR, FIS Optimization Algorithms), advanced feature extraction/selection (SFS, SFFS).
SA + Desktop PC	32–64 bit/ 700 MHz/Mb	Fast, medium size, portable, huge data capacity, consume not critical, expensive, not portable	Any / Visual	Linear, complex learning algorithms (GA, NeuroFuzzy Systems, mixture models, FIS Optimization Algorithms), advanced feature extraction/selection (SFS, SFFS), *etc.*

μC, microcontroller; μP, microprocessor; PIC, peripheral interface controller; SA, sensor array; NN, nearest neighbor algorithm; DSP, digital signal processing; KNN, k-nearest neighbor algorithm; PCA, principal component analysis; LDA, linear discriminant analysis; PCR, price coupling of regions; PLS, projection to latent structures; GA, genetic algorithm; APR, annual percentage rate; FIS, fuzzy inference system; SFS, shape from shading; SFFS, sequential forward floating selection.

**Table 3.** Summary of advantages and disadvantages of MOX and CP sensors, modified from [54].

**Sensor Type**	**Advantages**	**Disadvantages**
Metal-Oxide Semiconductor (MOX)	Very high sensitivity Limited sensing range Rapid response and recovery times for low mol. wt. compounds	High temperature operation High power consumption Sulfur & Weak acid poisoning Limited sensor coatings Sensitive to humidity Poor precision
Conductive Polymer (CP)	Ambient temperature operation Sensitive to many VOCs Short response time Diverse sensor coatings Inexpensive Resistance to sensor poisoning	Sensitive to humidity and temperature Sensors can be overloaded by certain analytes Sensor life is limited
